# Mechanisms of sodium channel clustering and its influence on axonal impulse conduction

**DOI:** 10.1007/s00018-015-2081-1

**Published:** 2015-10-29

**Authors:** Sean A. Freeman, Anne Desmazières, Desdemona Fricker, Catherine Lubetzki, Nathalie Sol-Foulon

**Affiliations:** 1 ICM-GHU Pitié-Salpêtrière, Sorbonne Universités UPMC Univ Paris 06, UMR_S 1127, 75013 Paris, France; 2 Inserm U1127, 75013 Paris, France; 3 CNRS UMR7225, 75013 Paris, France; 4 Assistance Publique-Hôpitaux de Paris, Hôpital Pitié-Salpêtrière, Paris, France

**Keywords:** Voltage-gated sodium channel, Node of ranvier, Axon–glial interactions, Myelin, Action potential propagation, Neurological disease

## Abstract

The efficient propagation of action potentials along nervous fibers is necessary for animals to interact with the environment with timeliness and precision. Myelination of axons is an essential step to ensure fast action potential propagation by saltatory conduction, a process that requires highly concentrated voltage-gated sodium channels at the nodes of Ranvier. Recent studies suggest that the clustering of sodium channels can influence axonal impulse conduction in both myelinated and unmyelinated fibers, which could have major implications in disease, particularly demyelinating pathology. This comprehensive review summarizes the mechanisms governing the clustering of sodium channels at the peripheral and central nervous system nodes and the specific roles of their clustering in influencing action potential conduction. We further highlight the classical biophysical parameters implicated in conduction timing, followed by a detailed discussion on how sodium channel clustering along unmyelinated axons can impact axonal impulse conduction in both physiological and pathological contexts.

## Introduction

Electrical axonal propagation of the action potential (AP) leads to chemical neurotransmission through synapses, which drive important nervous system functions such as motor output, control of visceral organs, encoding of sensory stimuli, and higher order cognitive processing. Synaptic input received by the neurons is integrated in the somatodendritic region [1] and the initiation of the AP occurs at a region called the axon initial segment (AIS) [2]. The AIS is enriched in voltage-gated ion channels, particularly voltage-gated sodium channels (Na_v_) that permit the entry of depolarizing current in the form of Na^+^ ions [2, 3]. This depolarizing current will then passively spread along the next segment of axonal membrane downstream of the active region, while efflux of K^+^ from voltage-gated potassium channels (K_v_) in the regions trailing the AP will inactivate Na_v_ and slowly bring the patch of axonal membrane back to its resting potential [2, 3].

**Fig. 1:**
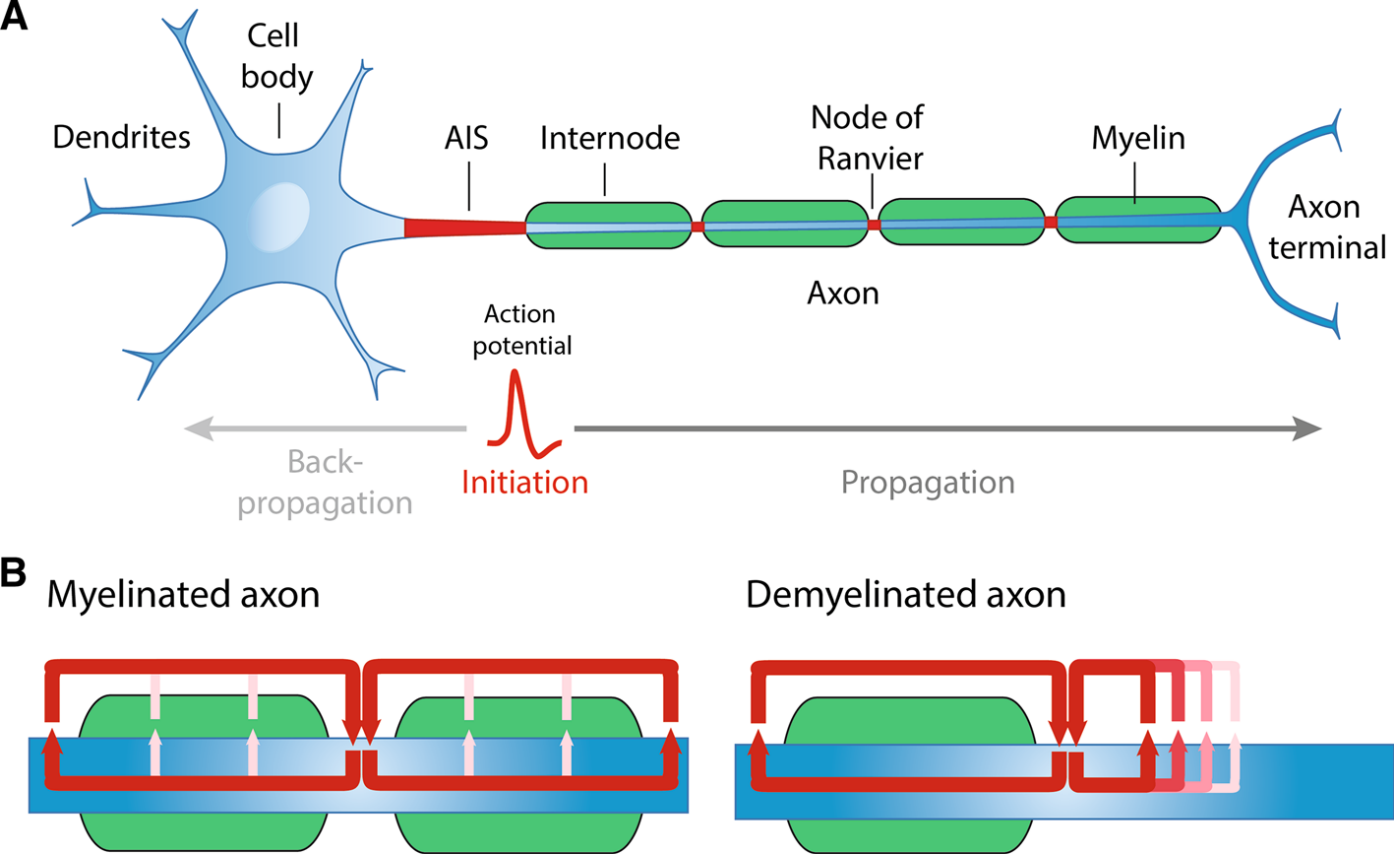
**a** Structural organization of a myelinated neuron and representation of action potential initiation and propagation. **b** In myelinated fibers, the action potentials are regenerated at the nodes of Ranvier, where high membrane currents (indicated by the *red arrows*) are observed. In case of demyelination, the propagation of action potentials is slowed down or blocked

Once the AP is triggered, it propagates down the axon to reach the synaptic terminals to relay information to the next neuron and carry out proper nervous system functions. Certain neural functions require adjustment of conduction velocity to regulate the synchrony of inputs [4] and fast conduction velocity can be critical for defense and survival. Several biophysical parameters govern the speed by which APs are propagated: axonal diameter, sodium channel density and temperature [3]. In addition, myelination of axons, which is one of the last evolutionary steps in the nervous system of jawed vertebrates [5], ensures rapid propagation of action potentials. Myelin is a lipid-rich, multilamellar membranous structure produced by Schwann cells in the peripheral nervous system (PNS) and oligodendrocytes in the central nervous system (CNS). Myelin provides both insulation of electric current and metabolic support for the axon [6–8]. Importantly, between the segments of myelin are unmyelinated gaps highly enriched with Na_v_ channels, called the nodes of Ranvier, which permit the regeneration of the AP and fast AP propagation through saltatory conduction (Fig. 1a). In addition, studies have shown that clusters of Na_v_ along unmyelinated fibers, but also prior to myelination in fibers destined to be myelinated, can influence axonal impulse conduction [9–12]. In this review, we will outline the molecular architecture of the nodes of Ranvier and mechanisms underlying the clustering of Na_v_ channels in both the PNS and CNS. Further detail will be provided to how APs are propagated along unmyelinated and myelinated fibers, particularly how different biophysical properties of the myelin sheath can affect axonal conduction. Finally, we will describe how the clustering of Na_v_ channels along unmyelinated and myelinated fibers can regulate AP conduction in the physiological and pathological state.

## Molecular composition of the nodes of Ranvier

The nodes of Ranvier are macromolecular complexes of ~1 µm length that are highly enriched in voltage-gated sodium channels [6]. These channels consist of a heterotrimeric complex of a pore forming *α*-subunit of approximately 260-kDa, and two differing auxiliary *β*-subunits that can interact with the *α*-subunit through non-covalent interactions (*β*1Na_v_ and *β*3Na_v_) or covalent disulfide bonds (*β*2Na_v_ and *β*4Na_v_) [13, 14]. The *SCNA* family encodes for ten different sodium channel *α*-subunit isoforms [15], yet only Na_v_1.1, Na_v_1.2, Na_v_1.6, Na_v_1.7, Na_v_1.8, Na_v_1.9 have been reported to be clustered at the nodes [16, 17]. Mammalian sodium channel *β*-subunits form a family of five proteins (*β*1Na_v_, *β*1BNa_v_, *β*2Na_v_, *β*3Na_v_, and *β*4Na_v_) that are encoded by four *SCNB* genes, but only *β*1Na_v_, *β*2Na_v_, and *β*4Na_v_ are expressed at the nodes [14, 18–20]. Na_v_ *β*-subunits are described as “auxiliary” subunits, but they play critical roles in regulating the rate of *α*-subunit activation and inactivation [21, 22], regulating the amount of resurgent sodium current and sodium current density [23–25], and regulating the plasma membrane insertion of *α*-subunits [14, 19].

**Fig. 2:**
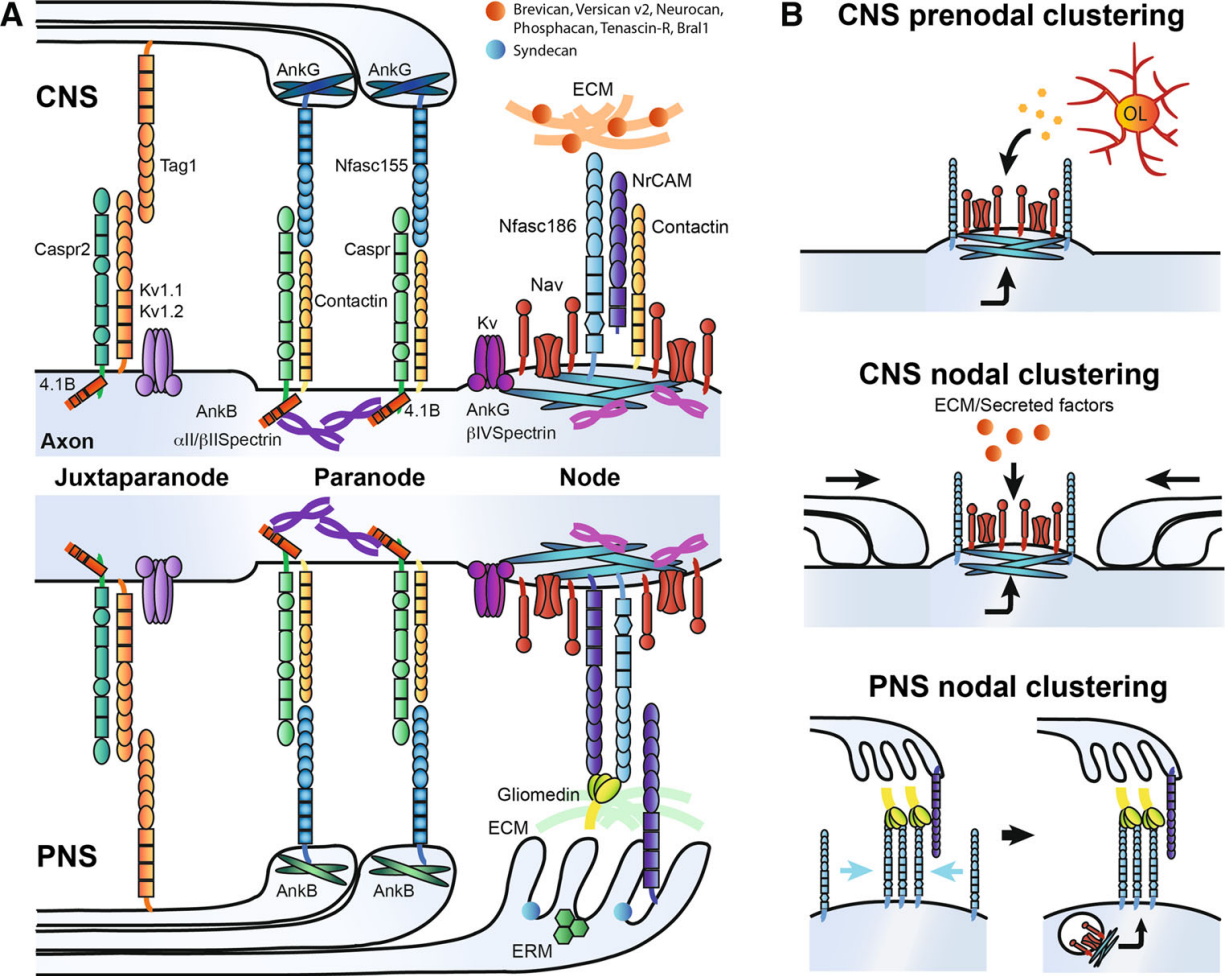
**a** Molecular organization of the nodes of Ranvier and surrounding domains in the CNS and PNS, respectively. **b** Mechanisms implicated in Na_v_ channel clustering at node-like clusters and nodes of Ranvier. Node-like clustering depends on both intrinsic and extrinsic cues (oligodendroglial-secreted factors). Nodal clustering differs in the CNS and the PNS. In the CNS, three different components (paranodes, extracellular matrix and scaffold proteins) play a role in Na_v_ channel assembly. In the PNS, early clustering of Nfasc186 through its interaction with glial Gliomedin and NrCAM is followed by targeting of scaffold proteins and voltage-gated channels

In addition to Na_v_ channels, K_v_ channels, cell-adhesion molecules (CAMs), cytoskeletal scaffolding proteins, and extracellular matrix (ECM) components are further clustered at the nodes of Ranvier (Fig. 2a) [6, 16]. The K_v_ channels K_v_7.2, K_v_7.3, and K_v_3.1b are responsible for the repolarization of the axonodal membrane and regulation of axonal excitability [26–30]. The L1-CAMs neurofascin186 (Nfasc186) and NrCAM are enriched at the nodes of Ranvier both in the PNS and the CNS [31, 32]. In addition, the glycosyl phosphatidylinositol (GPI)-anchored CAM contactin-1 is expressed at nodes only in the CNS [33, 34]. Interaction of Nfasc186, NrCAM or contactin-1 with the Na_v_ β1-subunit through their extracellular immunoglobulin-like domain may increase functional Na_v_ channel expression [18, 34, 35]. On the intracellular side of the nodal axolemma, Na_v_, Nfasc 186 and NrCAM can bind to the cytoskeletal scaffolding protein ankyrinG, which in turn binds with high affinity to βIV-spectrin, providing a link with the actin cytoskeleton [6, 16, 36]. The glycoprotein-rich ECM forms a negatively charged complex surrounding nodes of Ranvier that is involved in cation buffering and proper stabilization of the nodes [37–41]. Differentiating the PNS and CNS is the presence of Schwann cell microvilli that invade into the PNS nodal extracellular space and secrete several important molecules such as gliomedin and NrCAM, which together play a key role in clustering PNS nodes of Ranvier (see below; [42–44]). Although Schwann cell microvilli are absent from CNS nodes of Ranvier, CNS nodes are occasionally contacted by perinodal astrocytes, but their function at these domains remains to be elucidated [45].

The regions flanking the nodes are the paranodes, where axoglial junctions between myelin loops and the axon form a ternary complex via interactions between axonal Caspr/contactin and glial Nfasc155 (Fig. 2a) [31, 46–50]. Importantly, the paranodes act as a molecular sieve to restrict the diffusion of nodal components [51]. Cytoskeletal scaffolding proteins are also enriched at paranodes in both myelinating glia and axons. Glial expression of the 190- and 270-kDa isoforms of ankyrinG is enriched in the CNS paranodes, while the 220-kDa isoform of ankyrinB is clustered at PNS paranodes [52, 53]. Loss of these glial proteins results in a delay of paranodal junction formation [52]. Axonal expression of cytoskeletal scaffolding components in both the CNS and PNS paranodes includes 4.1B, *α*II-spectrin, and ßII-spectrin, which are implicated in the organization and maintenance of the paranodal junction [53–58]. Recently, the K_v_ channel Slo/BK has been reported to cluster at the paranodes of rodent cerebellar Purkinje neurons, and its clustering at the paranodes is necessary for supporting high-frequency firing that is characteristic of cerebellar Purkinje neurons [59].

Another important function of the paranodes is to also act as a segregation barrier between the nodes and the juxtaparanodal regions that are highly enriched with K_v_1 [49, 57, 60, 61]. These K_v_ channels receive little depolarizing current since they are underneath the multiple layers of compacted myelin. The clustering of K_v_1 to the juxtaparanodal regions depends on the cell-adhesion molecules TAG-1 (expressed on both the glial and axonal sides) and axonal Caspr2 [62–64]. Underneath this region is a cytoskeletal complex composed of PSD-93/95, ADAM22, ßII-spectrin, *α*II-spectrin, and 4.1B [16]. The latter protein, 4.1B, plays an essential role in assembling the juxtaparanodal complex [54].

## Mechanisms of nodes of Ranvier assembly

Even though the PNS and CNS nodes of Ranvier have only little changes in their molecular composition, the mechanisms underlying their formation are not identical. This dissimilarity between the two nervous systems mainly stems from the differences in cell types producing the myelin sheath that ultimately govern the neuron–glia interactions necessary to form the nodes of Ranvier.

### Na_v_ clustering at node of Ranvier in the PNS

During initial myelination of the axon by the Schwann cell, components of the nodal complex cluster to regions adjacent to the myelinating segment called heminodes [65]. The initial steps of heminodal formation, which ultimately gives rise to mature nodes from the fusion of two heminodes at the edges of elongating myelin segments, depend on the interactions between axonal Nfasc186 and gliomedin and NrCAM emanating from Schwann cell microvilli (Fig. 2b) [42–44, 66–69]. This cooperative interaction is highlighted by the fact that Nfasc186 fails to properly localize to heminodes in both *NrCAM*- and *Gliomedin*-deficient mice; however, full nodes of Ranvier ultimately form in these knockouts through paranodal junctions acting as a restriction barrier to assemble the nodal components [44, 70]. For the heminodal assembly, glial NrCAM stabilizes secreted trimerized gliomedin for increased interaction with Nfasc186 [43, 44]. The expression of certain molecules of the basal lamina, microvilli, and ECM also appears to be involved in promoting the heminodal clustering mechanism [71–73]. Then, Nfasc186 plays an important role in nucleating and incorporating the nodal components to the axolemma (Fig. 2b). Nfasc186 interacts with ankyrinG, which in turn is able to recruit Na_v_ channels and its scaffolding partner βIV-spectrin [66, 67, 74, 75]. Axoglial contact mediated by gliomedin, NrCAM and Nfasc186 also contributes to the long-term maintenance of Na_v_ channels at nodes of Ranvier [76].

### Na_v_ clustering at node of Ranvier in the CNS

In contrast to the PNS, the molecular mechanisms underlying nodal assembly in the CNS are still partly understood. Three complementary mechanisms have been established to participate in CNS nodal assembly: ECM-induced cell-adhesion molecule clustering, scaffolding molecules anchoring the nodal complex to the actin cytoskeleton, and paranodal barrier formation (Fig. 2b) [41]. However, their relative importance and whether differences exist between neuronal subpopulations is still debated. In contrast to Schwann cells, oligodendrocytes do not contact nodes directly and do not express gliomedin. Other glia-derived ECM proteins (i.e., chondroitin sulfate proteoglycans, tenascin-R, Bral1) form complexes with axonal CAMs such as Nfasc186, NrCAM, contactin-1 and the β-subunits of sodium channels [37–39, 77, 78]. Yet, the clustering of ECM molecules at the nodes of Ranvier in vivo occurs after the clustering of axonodal components in the mouse optic nerve [41], and nodal assembly still occurs in mutant mice lacking these ECM proteins, suggesting that ECM molecules may be involved in the stabilization of the nodes rather than initiating their assembly [37, 39–41, 77].

Axonodal CAMs contribute to the assembly of CNS nodes of Ranvier. Transgenic expression of axonal Nfasc186 or Nfasc140 in *Nfasc*-*null* mice, which form neither nodes nor paranodes, rescues Na_v_, CAMs, ECM and cytoskeletal components of the CNS nodes of Ranvier [61, 79]. In addition, loss of the CNS nodal and paranodal GPI-anchored protein Contactin-1 results in reduced numbers of nodes of Ranvier [80].

The paranodal barrier formed through direct axoglial contacts established at the paranodal junctions also participates in the assembly of CNS nodes of Ranvier [41, 81]. In this context, in *Nfasc* null mice, the reconstitution of paranodes by glial expression of Nfasc155 is sufficient to rescue Na_v_ channel clustering [61]. Double knockout mice for βIV-spectrin and ECM components (thereby leaving the paranodal junctions intact) can still assemble CNS nodes of Ranvier, albeit with reduced Na_v_ clustering compared to wild-type and single knockout mice [41]. However, other studies have shown that the timing or number of developing nodes of Ranvier is unaffected by either suppressing the paranodes through inactivation of genes coding for the paranodal constituents Caspr and Nfasc155 or by disrupting the paranodal junctions through loss of myelin proteins or lipids [49, 61, 82–85]. Overall, these results suggest that, while paranodal junctions have the ability to cluster CNS nodes of Ranvier, they might not be essential for CNS nodal assembly. Conversely, paranodal junctions are particularly important for nodal maintenance, suggesting that mechanisms of nodal stabilization depend on protein–protein interactions that are different from those that dominate initial assembly [51, 83, 86].

CNS nodes of Ranvier can also be assembled through intrinsic neuronal mechanisms directed by axonal scaffolding proteins such as ankyrinG. AnkyrinG is able to bind several membrane-spanning axonodal proteins through its multiple ANK repeats and connects them to the neuronal actin cytoskeleton [87], thereby laying the foundation for a large heterogeneous macromolecular complex at the nodes. The importance of ankyrinG in CNS nodal assembly is highlighted by the fact that loss of the giant 270- and 480-kDa ankyrinG splice variants results in a significant reduction in CNS nodal formation [88]. However, it has also been reported that erythrocyte ankyrin, ankyrinR, can substitute for ankyrinG when ankyrinG is completely lost [89]. AnkyrinG also plays an important role in trafficking Na_v_ to the nodes via its direct interaction with the conventional anterograde microtubule motor kinesin-1 [90]. Taken together, these results point to ankyrinG as an important molecule that directs CNS nodes of Ranvier assembly through linking the nodal components together and through trafficking of Na_v_ channels.

Finally, axonal clustering of Na_v_ channels before myelin deposition and oligodendroglial contact has been shown to occur in retinal ganglion cell cultures, where these clusters were induced by oligodendroglial-secreted factor(s) [91, 92]. More recently, it has been shown that nodal-like clusters (i.e., clusters of Na_v_ channels colocalizing with the scaffold protein ankyrinG and nodal CAMs) are detected before myelin deposition along axons in hippocampal neuron-glia cultures and in the developing hippocampus in vivo. These clusters can be induced by oligodendroglial-secreted factor(s) and depend on ankyrinG for their assembly [11]. Importantly, nodal-like clusters are restricted to hippocampal GABAergic neurons, whereas clustering of nodal proteins along the axons of hippocampal pyramidal neurons occurs concomitantly with myelin ensheathment, suggesting separate mechanisms of assembly among different regional neuronal subpopulations [11].

Clustering of Na_v_ channels will eventually influence AP propagation along axons and the specific role of their clustering, as well as the classical biophysical parameters implicated in conduction velocity will be further highlighted.

## Action potential initiation and propagation along unmyelinated fibers

The preferred site of AP initiation is the distal end of the AIS [93, 94], where the density of low-threshold Na_v_1.6 sodium channels is highest [95]. During AP initiation, the active depolarization backpropagates to the soma (antidromic) and down the axon (orthodromic) [96, 97]. Propagation of the AP along the axon is dependent on how fast the membrane is able to charge, which is determined by the membrane capacitance (how much charge is stored on the axonal membrane per unit area) and the axial resistance (how resistant the interior axonal medium is to electrical current). Factors involved in how quickly the membrane will charge, and consequently increasing conduction velocity, are characterized by either a reduction in the membrane capacitance or a reduction in the internal axial resistance [98]. The reduction in the internal axial resistance may be achieved through increasing the diameter of the axon, which reduces the resistivity Na^+^ ions must face as they passively spread through the axoplasm. Accordingly, the AP conduction velocity in unmyelinated axons is generally described from theoretical calculations to be proportional to the square root of the axonal diameter [99, 100].

Conduction velocity along unmyelinated vertebrate CNS axons can be measured electrophysiologically using dual soma-axon patch clamp recordings in pyramidal neurons in acute brain slices [94], or by patching axon “blebs” where investigators patch both the soma and the cut end of the axon in pyramidal cells or interneurons [101–106]. Orthodromic conduction velocities along these axons ranged from 0.2 to ~1.45 ms^−1^ (for review see [107]).

## Regulation of action potential propagation along myelinated fibers

While increasing axonal diameter is a viable solution for rapid axonal conduction, it also comes at a price in terms of space constraints and energetics [108]. Maintaining the extracellular Na^+^ and intracellular K^+^ ion gradient, mediated through the action of Na^+^/K^+^ pumps, is energetically costly [109], even though ion channel kinetics underlying the AP in pyramidal neurons are built to be energy-efficient and to minimize the overlap of Na^+^ and K^+^ ion fluxes [110]. Cerebellar Purkinje neurons and fast-spiking interneurons, however, have incomplete inactivation of sodium channels leading to reduced metabolic efficiency [111].

Myelination permits optimization of fast axonal AP propagation over long distances. The insulating properties of the myelin sheath reduce the leakage of Na^+^ current that flows down the axon (i.e., increase in axial resistance) and reduce the axonal capacitance in conjunction with nodes of Ranvier, thereby allowing for faster charging of the axonal membrane [98]. Myelination is generally beneficial in increasing conduction velocity compared to the unmyelinated nerve when the CNS axonal diameter is above 0.2 µm [112]. Indeed, this fact correlates well with the finding that only axons with a diameter > 0.2 µm can be myelinated in the CNS [112]. The speed of nervous conduction in myelinated axons is linearly proportional to the axonal diameter [99, 113], partly due to increased myelin thickness [113, 114].

Together with axonal diameter, the thickness of the myelin sheath plays an important role in speed of axonal AP propagation. Typically, myelinated axons are classified by their g-ratio, which is a calculation of the ratio between the axonal diameter to the overall diameter of the fiber, and this ratio is optimized (g-ratio between 0.6 and 0.77) to ensure maximal conduction and efficiency [99, 115]. The regulation of myelin thickness is highly important in maintaining this optimum since hypermyelination, such as that observed in the absence of Dlg1-PTEN in peripheral nerves, can lead to unstable myelin sheaths which may ultimately attenuate nerve conduction [116]. The paranodal junctions are key determinants in maintaining rapid axonal conduction through their barrier-like seals that form between the paranodal loops of the myelinating glial cells and the axolemma. These junctions are important for restricting the short-circuiting of the nodes of Ranvier [117] and for metabolic savings for AP regeneration [98].

Conduction timing along myelinated neurons in the CNS ranges from modestly fast to rapidly conducting [107]. Utilizing the dual patch soma-bleb technique or rapid acquisition of voltage changes through voltage-sensitive dyes reports of conduction speeds in pyramidal neurons of the cortex range from ~0.5 to 4.5 ms^−1^ [96, 97, 118, 119]. Conduction speeds along Purkinje cell axons obtained from bleb recordings and more recently from extracellular antidromic axonal recordings range between 0.52 and 0.77 ms^−1^ [59, 120]. These values are not much faster compared to those of the unmyelinated axons in the brain, most likely related to the small diameters of these axons. Indeed, in larger diameter axons, such as in motoneurons and along adult mouse sciatic nerve, action potential conduction can reach up to 80 ms^−1^ [121, 122]. Differences and lower speeds of conduction in the cortex could be related to the fact that these neurons may need to synchronize conduction timing for proper cortical circuit activity, and also that these neurons may be maximizing their metabolic energetic needs in preference to a further increase in axonal conduction.

## Na_v_ channel clustering regulates axonal conduction

### Concentration of Na_v_ channels and size and structure of the nodes of Ranvier

Not only are axonal diameter and myelin sheath thickness crucial for increasing conduction velocity, but also the density of Na_v_ channels. A recent study has shown that along unmyelinated basket cell hippocampal axons, Na_v_ conductance density increases tenfold in a gradient-wise manner from the soma (2.6 channels/µm^2^) towards the proximal axon (25 channels/µm^2^ in the proximal axon), followed by a further increase in Na_v_ density at the distal axon (46.1 channels/µm^2^) [105]. This increase in distal Na_v_ channels ensures a supercritical density necessary for supporting fast AP propagation along the axons of these fast-spiking GABAergic neurons [105]. In myelinated fibers, however, Na_v_ is highly clustered at the nodes of Ranvier in the order of ~1200 channels/µm^2^ [123], while internodes contain ~20–25 channels/µm^2^ [124]. Theoretical and experimental studies show that this asymmetrical concentration of Na_v_ between the nodes of Ranvier and the internodes reduces the axonal capacitance and the concentration of Na^+^ necessary to regenerate the AP, resulting in improving energy efficiency [9, 125, 126]. Moreover, ECM glycoproteins surrounding the nodes of Ranvier can increase conduction velocity through concentrating Na^+^ in the vicinity of Na_v_ channels [37, 40]. Another advantage of this restriction of high density of Na_v_ at the nodes of Ranvier is that it permits high-fidelity impulse conduction because the ratio of current available to stimulate the node to the current necessary to depolarize the node, otherwise called the safety factor, is very high (in the order of 5–7). When the safety factor is less than 1, such as in the case of drastic changes in axonal geometry and also in demyelinating pathologies, conduction block occurs and the AP fails to propagate [3].

A second component related to the nodal capacitance is the membrane area occupied by the node of Ranvier. Increasing the size of the nodes of Ranvier can alter conduction velocity, such that an increase in nodal length would result in slowing of the AP being regenerated at the adjacent node due to increased nodal capacitance. Experimental evidence in the electric organ of *Sternarchus* showed that large nodes of Ranvier have delayed conduction compared to smaller nodes of Ranvier [127]. A recent theoretical study has also demonstrated that conduction velocity decreases when the nodal width is increased [128]. In addition, fenestrated nodes and the wide submyelinic space form the basis for unusually fast impulse conduction in shrimp giant nerve fibers that display remarkable conduction speeds of more than 200 ms^−1^, making them among the fastest-conducting fibers recorded [129].

### Sodium channel isoforms

The biophysical gating properties of the Na_v_ subunits allow for several types of current flow through the pore of the Na_v_ *α* -subunit, and can therefore support several types of neuronal AP firing properties, which may affect AP conduction. At the AIS of cortical pyramidal neurons, two Na_v_ channel isoforms are asymmetrically distributed: Na_v_1.2 is enriched in the proximal part of the AIS and it has a high threshold for activation. The low-threshold Na_v_1.6 accumulates at the distal end, and therefore, favors spike initiation at this region [95]. At the nodes of Ranvier, several different Na_v_ channel isoforms cluster at varying developmental stages [11, 92, 130, 131], and the relative contribution of the various nodal ion channels on axonal conduction is poorly understood. Our understanding of the roles of Na_v_ isoforms in relation to AP conduction is currently limited to electrophysiological studies investigating the biophysical gating and current dynamics of the nodal Na_v_ isoforms. Na_v_1.2, which is primarily associated to the mammalian immature PNS and CNS nodal-like clusters and nodes of Ranvier [11, 92, 130, 132, 133] needs large depolarizing current to fire and inactivates during high-frequency firing [134]. Na_v_1.6 and Na_v_1.1, which are clustered to mature nodes of Ranvier and nodal-like clusters in the PNS and CNS [11, 130, 131, 133, 135], participate in persistent sodium current [134, 136, 137]. This slowly inactivating current may therefore drive faster conduction through the increase of axoplasmic Na^+^ [134, 137–139]. With their low-threshold voltage dependence, Na_v_1.6 may favor spike initiation not only in the AIS but also ectopically, at the nodes. Na_v_1.6 rapidly recovers from inactivation and may also sustain high rates of activity [140, 141]. These data suggest that the sodium channel subunit composition at nodes of Ranvier may contribute to a high safety factor for AP propagation fidelity [107]. Nevertheless, further tools need to be developed to understand the different contributions of each nodal Na_v_ isoform on AP conduction.

### Number of nodes and internodal length

Theoretical and experimental evidence suggests that myelinated axons have an optimal internodal length for maximal conduction, but changes in the internodal length may have modest to drastic consequences to conduction velocity based on their shortening or lengthening [113, 114, 142–148]. Court and colleagues (2004) showed that, in *periaxin*-*null* mice where Schwann cell elongation fails, thereby exhibiting decreased internodal length but normal axon diameter and myelin thickness, conduction velocity along these fibers was significantly decreased, suggesting that conduction velocities are highly sensitive to substantially shorter internodal lengths [143]. Shortened internodal lengths, however, may be physiologically relevant in synchronizing conduction timing of different inputs [149]. In the avian auditory brainstem, nucleus magnocellaris cells in the avian cochlear nucleus have both ipsilateral and contralateral inputs to the nucleus laminaris, which is responsible for the processing of auditory information. Challenges in synchronizing aural input arise when taking the length difference of ~ 1600 µm between the contralateral and ipsilateral axonal branches from nucleus magnocellaris—yet, proper simultaneous timing of input is ensured by the ipsilateral projections having shorter internodes and a smaller axonal diameter than that of the contralateral projections [149]. Thus, in the case of the avian auditory system, shortened internodes act as a physio-anatomical way of slowing down AP conduction and therefore secure correct encoding of auditory information from bilateral inputs [149, 150]. However, recent work from Ford et al., in analogous mammalian auditory brainstem circuits, reports that axons responding to low-frequency sounds had a larger diameter but shorter internodes than high-frequency axons, and higher conduction velocities. Moreover, internode length decreased and nodes of Ranvier diameter increased progressively along the distal axon segments, which simulations predict was important to adjust precisely the conduction velocity of APs within the circuit [148].

Although significant shortening of the internodes gives rise to substantial variations in AP conduction velocity [143, 144], lengthening of the internodal region appears to have only modest effects on conduction velocity at intermediate lengths [98, 144]. Experimental evidence correlates well with these theoretical studies. Mutant mice lacking the N-terminal PDZ domain of periaxin delays Schwann cell elongation and results in shorter internodes and lowered conduction velocities compared to controls at 3 and 6 weeks of age [145]. However, at 16 weeks of age, conduction velocities are indistinguishable between mutant and wild-type mice even though internodal lengths are significantly different, suggesting that conduction velocity speeds reach a “flat maximum” once the distance between nodes have reached a certain threshold. Simpson and colleagues further showed that by increasing internodal length by up to 35 % and keeping axon diameter and myelin sheath thickness constant, there was no significant increase in axonal conduction speeds [146]. Together, these theoretical and experimental data suggest that increases in internodal distance above the optimum result in insignificant changes in axonal impulse conduction.

Finally, a recent work from Tomassy et al. has described a new pattern of myelin distribution along single axons, where myelinated segments are interspaced with long, unmyelinated tracts, in some pyramidal neurons in the neocortex [151]. Although the functional consequences of these heterogeneous profiles of myelination await further identification, the profile of longitudinal distribution of myelin may have evolved as a strategy to modulate long-distance communication in the neocortex [151].

### Na_v_ clustering along unmyelinated axons

Several reports have observed nodal-like clusters of Na_v_ along unmyelinated fibers. These have been reported in both the PNS and CNS fibers in a number of different species including the marine invertebrate *Aplysia* [12], zebrafish mutants lacking Schwann cells in peripheral nerves [152], rodent retinal ganglion cells [85, 86] and hippocampal GABAergic interneurons [11, 91, 92], in dorsal and ventral spinal roots of dystrophic mice lacking merosin [153], in lipid rafts of group-C nerve fibers [154], and unmyelinated axonal segments in non-pathological human dental pulp [155]. In light of these qualitative observations, what could be the possible functional role of these dense aggregates of Na_v_ along unmyelinated axons?

Few studies have attempted to assess the physiological role of these focal Na_v_ clusters. Waxman et al. (1983) using transmission and freeze-fracture electron microscopy proposed that the electron-dense subaxolemmal particles along the axons of the nerve fiber layer of the adult rat retina could correspond to clusters of Na_v_ that act as electrogenic “hot-spots” [156, 157]. Further theoretical work by Johnston and colleagues (1996) modeling their observation of Na_v_ clusters along cultured and ganglion neurons from *Aplysia* showed that the clustering of Na_v_ along unmyelinated axons required 30–60 % fewer channels to propagate APs compared to an evenly diffuse expression of Na_v_, suggesting that the physical clustering of these channels acts as a way of optimizing AP conduction [12]. Recent work by Freeman et al. also suggests that these clusters are associated with increased AP conduction. Through simultaneous soma-axon electrophysiological recordings, they observed that nodal-like clusters along GABAergic axons increase conduction velocity by 150 % in comparison to GABAergic axons without clusters [11]. Importantly, this increase is observed independently of axonal caliber, representing a novel means for accelerated axonal propagation of APs before myelin deposition [11]. This augmentation in AP conduction may also underlie what was observed by Foster and colleagues (1982) when they reported that during rat optic nerve development there was an increase in AP conduction prior to myelination that could not be accounted by an increase in axonal caliber [158]. Moreover, experimental and theoretical findings suggest that micro-saltatory conduction may occur in the absence of myelination due to the dense clustering of Na_v_ at nodal-like domains [9, 10, 154]. Lastly, the action of Na_v_ clustering along unmyelinated axons may be important for overcoming axonal branch point failures and maintaining reliable propagation of APs [3]. In this respect, theoretical modeling calculations predict that high-density sodium channel clusters could serve as acceleration points [159] and therefore Na_v_ clustering in the absence of myelination could be a way of maintaining faithful propagation of the AP.

### Pathologies leading to alterations of Na_v_ clustering and conduction velocity

Demyelination or alteration of myelin, disruption of paranodal junctions or primary nodal impairment, leading to abnormal ion channel expression and lengthening or disruption of the node of Ranvier may contribute to altering the conduction along myelinated axons in several diseases, including stroke, spinal cord injury, multiple sclerosis (MS), and Guillain–Barré syndrome (GBS) (for review see, [109, 160–162]).

Various dysmyelinating mutant animal models such as Trembler-J mice, characterized by a mutation in the peripheral myelin protein 22 gene, and Shiverer mice, which have a disrupted myelin basic protein gene MBP, have improperly formed and distributed paranodes and abnormal Na_v_ clusters [81, 163]. Similarly, in demyelinated lesions of MS or experimental autoimmune encephalomyelitis (EAE), an animal inflammatory model for MS, a disruption of nodal, paranodal and juxtaparanodal domains has been reported, replaced by diffuse expression of the components of these domains along the denuded portion of the axon [164–167]. More current is required to drive depolarization of the axolemma, and this raises the metabolic cost of maintaining the Na^+^/K^+^ gradient. Moreover, the failure of K_v_1 to be clustered at the juxtaparanodes or their mislocalization to either the paranodes or nodes has an important impact on axonal conduction [168]. Diffusion of nodal markers is accompanied by a switch from the mature Na_v_1.6 to the immature Na_v_1.2 isoform, which could be an adaptive response, since Na_v_1.6 might favor axonal damage by inducing persistent current that will drive the inversion of the Na^+^/Ca^2+^ exchanger and subsequent calcium-mediated axonal damage [165]. Altogether these changes can lead to conduction failures that manifest into substantial neurological deficits in MS patients (Fig. 1b). Axoglial organization is fully restored after remyelination, as observed in shadow plaques in tissue taken from MS patients [164]. Furthermore, clustering of Nav channels has been observed on PLP-negative (i.e., non-remyelinated) fibers within lesions undergoing remyelination [164], suggesting that, as observed for developmental myelination, nodal protein clustering might precede myelin repair. Although the mechanisms of axonal domain reassembly during remyelination are still poorly understood, it can be hypothesized that, similar to early nodal clustering during developmental myelination, these clusters may accelerate conduction velocity before remyelination and therefore participate in functional recovery. The observation of saltatory conduction occurring before remyelination in axons demyelinated with lysophosphatidylcholine may also support this hypothesis [169]. A reduction of the internodal length is observed after remyelination, which may induce changes in conduction velocity but may also have a negative impact due to the increase in the energy needed for AP propagation [109].

Nodal and perinodal proteins are also direct targets of autoimmune reactions, and autoantibodies or specific T cells can be detected notably in patients with MS (reviewed in [160, 161]). These autoantibodies or T cells, administered to the animals with EAE, can induce pathogenic effects such as acute axonal injury [170, 171]. Similarly, in GBS, an acute autoimmune polyradiculoneuropathy, the autoimmune processes specifically target gangliosides (GM1 or GD1a or b) highly enriched at node, which is the site of primary injury. Autoantibodies to gliomedin, Nfasc or contactin have also been found in some patients with GBS. These antibodies can induce lengthening of the nodes of Ranvier and disruption of their molecular organization leading to conduction failure probably due to dysfunction of Na_v_ channels [161].

Axonal degeneration, depending on the specific disorder and its severity, eventually follows conduction block [162]. Indeed, defects in Na^+^/K^+^ pump function due to ATP depletion induce axoplasmic Na^+^ accumulation, which in turn reverses the Na^+^/Ca^2+^ exchanger to remove excess Na^+^. Consequently, Ca^2+^ accumulation may activate calpain, a protease capable of inducing proteolytic cleavage of neurofilaments, mitochondrial damage and Wallerian degeneration [162].

## Conclusion

Beyond axon diameter and the presence of a myelin sheath, Na_v_ channel nodal clustering is also a key process regulating AP propagation along axons. Although the understanding of molecular mechanisms that support nodes of Ranvier assembly and maintenance in physiological conditions is progressing, some questions remain to be clarified regarding the diversity of neuronal responses during development and nodal reassembly after a lesion. An attractive hypothesis is that nodal-like cluster formation on unmyelinated axons might be associated with the need for early establishment of neuronal connections during development on axons with long trajectories. Whether nodal-like cluster formation on unmyelinated fibers will initiate myelination, and how cross-talk between glial cells and specific neuron subpopulations regulates axonal transmission, raise an exciting field of research. Recent findings showing non-uniform myelin distribution along single axons in the neocortex of adult mice, underlie differences in myelination profiles, which is an integral feature of neuronal identity [151]. This suggests further levels of conduction velocity regulation and a possible plasticity in network behavior.

## Abbreviations

AP: Action potential
AIS: Axon initial segment
Na_v_: Voltage-gated sodium channels
K_v_: Voltage-gated potassium channels
PNS: Peripheral nervous system
CNS: Central nervous system
CAM: Cell-adhesion molecule
ECM: Extracellular matrix
Nfasc: Neurofascin
GPI: Glycosyl phosphatidylinositol
MS: Multiple sclerosis
GBS: Guillain–Barré syndrome
EAE: Experimental autoimmune encephalomyelitis
